# Supplementary material for: Iterative improvement in the automatic modular design of robot swarms
Source: PeerJ Comput Sci. 2020 Dec 7;6:e322. doi: 10.7717/peerj-cs.322 (PMC7924708; doi:10.7717/peerj-cs.322)
Supplement: Supplemental Information 3 [file peerj-cs-06-322-s003.zip › argos3/doc/api/standalone/a00575_source.html]

ARGoS: plugins/simulator/entities/led\_equipped\_entity.h Source File


- Main Page
- Related Pages
- Namespaces
- Classes
- Files

- File List
- File Members

# plugins/simulator/entities/led\_equipped\_entity.h

Go to the documentation of this file.

```
00001 
00007 #ifndef LED_EQUIPPED_ENTITY_H
00008 #define LED_EQUIPPED_ENTITY_H
00009 
00010 namespace argos {
00011    class CLEDEquippedEntity;
00012    class CLEDEntity;
00013 }
00014 
00015 #include <argos3/core/simulator/entity/composable_entity.h>
00016 #include <argos3/plugins/simulator/entities/led_entity.h>
00017 #include <map>
00018 
00019 namespace argos {
00020 
00036    class CLEDEquippedEntity : public CComposableEntity {
00037 
00038    public:
00039 
00040       ENABLE_VTABLE();
00041 
00042       typedef std::map<std::string, CLEDEquippedEntity*> TMap;
00043 
00044    public:
00045 
00046       struct SActuator {
00047          typedef std::vector<SActuator*> TList;
00048 
00049          CLEDEntity& LED;
00050          CVector3 Offset;
00051          SAnchor& Anchor;
00052 
00053          SActuator(CLEDEntity& c_led,
00054                    const CVector3& c_offset,
00055                    SAnchor& s_anchor);
00056       };
00057 
00058    public:
00059 
00064       CLEDEquippedEntity(CComposableEntity* pc_parent);
00065 
00071       CLEDEquippedEntity(CComposableEntity* pc_parent,
00072                          const std::string& str_id);
00073 
00077       ~CLEDEquippedEntity();
00078 
00079       virtual void Init(TConfigurationNode& t_tree);
00080 
00081       virtual void Reset();
00082 
00083       virtual void Enable();
00084 
00085       virtual void Disable();
00086 
00093       void AddLED(const CVector3& c_offset,
00094                   SAnchor& s_anchor,
00095                   const CColor& c_color = CColor::BLACK);
00096 
00106       void AddLEDRing(const CVector3& c_center,
00107                       Real f_radius,
00108                       const CRadians& c_start_angle,
00109                       UInt32 un_num_leds,
00110                       SAnchor& s_anchor,
00111                       const CColor& c_color = CColor::BLACK);
00112 
00119       CLEDEntity& GetLED(UInt32 un_index);
00120 
00126       inline SActuator::TList& GetLEDs() {
00127          return m_tLEDs;
00128       }
00129 
00136       inline const CVector3& GetLEDOffset(size_t un_idx) const {
00137          ARGOS_ASSERT(un_idx < m_tLEDs.size(),
00138                       "CLEDEquippedEntity::GetLEDOffset() : index " <<
00139                       un_idx <<
00140                       " out of bounds [0:" <<
00141                       m_tLEDs.size()-1 <<
00142                       "]" );
00143          return m_tLEDs[un_idx]->Offset;
00144       }
00145 
00151       void SetLEDOffset(UInt32 un_index,
00152                         const CVector3& c_offset);
00153 
00159       void SetLEDColor(UInt32 un_index,
00160                        const CColor& c_color);
00161 
00167       void SetAllLEDsColors(const CColor& c_color);
00168 
00176       void SetAllLEDsColors(const std::vector<CColor>& vec_colors);
00177 
00183       void AddToMedium(CLEDMedium& c_medium);
00184 
00189       void RemoveFromMedium();
00190 
00191       virtual std::string GetTypeDescription() const {
00192          return "leds";
00193       }
00194 
00195    protected:
00196 
00197       virtual void UpdateComponents();
00198 
00199    protected:
00200 
00202       SActuator::TList m_tLEDs;
00203    };
00204 
00205 }
00206 
00207 #endif
```

---

Generated on 10 Jul 2018 for ARGoS by 
 1.6.1
